# Supplementary material for: Harnessing Ionic Interactions and Hydrogen Bonding for Nucleophilic Fluorination
Source: Molecules. 2020 Feb 7;25(3):721. doi: 10.3390/molecules25030721 (PMC7037423; doi:10.3390/molecules25030721)
Supplement: Supplementary file 1 [file molecules-25-00721-s001.pdf]

Harnessing ionic interactions and hydrogen bonding for nucleophilic fluorination

Young-Ho Oh,[1] Hyoju Choi,[1] Chanho Park,[2] Dong Wook Kim,[*2] Sungyul Lee[*2]

[1]*Department of Applied Chemistry, Kyung Hee University, Gyeonggi 17104, Republic of Korea.*

[2]*Department of Chemistry and Chemical Engineering, Inha University, Incheon 402-751, Republic of Korea.*

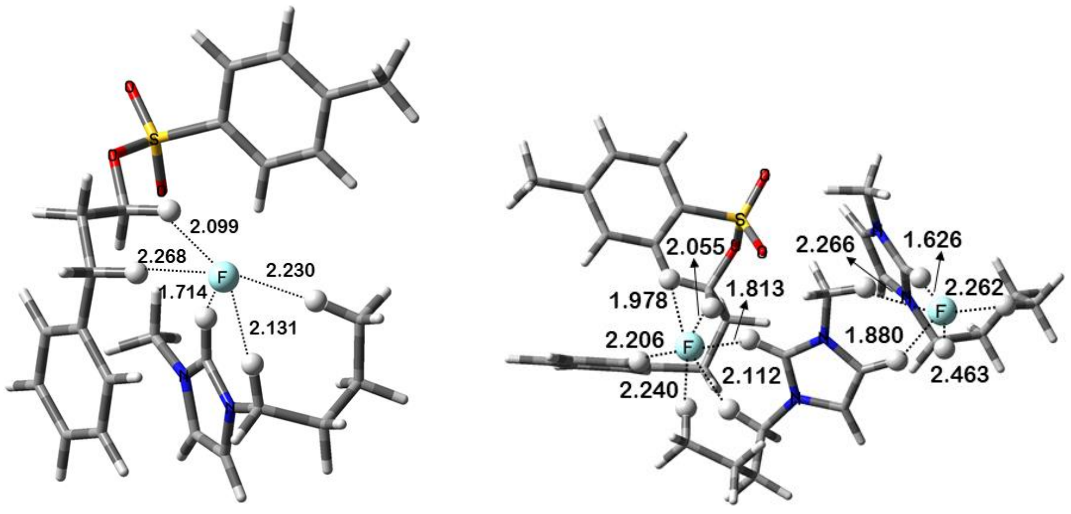

(a) (b)

Figure 2S. Pre-reaction complexes of S_N2 fluorination in [bmim][F]. (a) equivalent of IL = 1 (b) equivalent of IL = 2. M06-2X /6-311G** method for C, H, O, N, S, F atoms, using Gaussian 09 set of programs.

Cartesian coordinates for conformers

(a)
C -3.50080600 0.54880000 2.24363100
C -3.85966900 1.50099600 1.34373000
C -1.92626800 0.64698500 0.69670800
N -2.29439000 0.03294300 1.82108200
H -3.98672900 0.19322500 3.13678400
H -4.72554800 2.14004900 1.29786000
H -1.03905000 0.50579900 0.05430000
N -2.86558500 1.54167500 0.39317300
C -1.53980600 -1.01132500 2.50730600
H -1.37195100 -0.70858700 3.54045300
H -2.10227000 -1.94407300 2.47971400
H -0.57936000 -1.13853200 2.01164300
C -2.82040300 2.40775900 -0.79331600
H -1.92354700 2.09886000 -1.33673100
H -3.70273400 2.18375800 -1.39647900

C -2.78764400 3.88167500 -0.39915200
H -2.75949800 4.46124600 -1.32725600
H -3.72229500 4.14210200 0.10833100
C -1.59242500 4.25351000 0.47951400
H -1.69172200 5.30336800 0.77027800
H -1.63094500 3.67472900 1.40963100
C -0.24771000 4.03539900 -0.21312100
H 0.57431200 4.34088400 0.43913100
H -0.09324900 2.98953700 -0.49894000
H -0.18698900 4.63842700 -1.12500900
F -0.14349400 0.92679600 -1.34506300
C -2.30431600 -2.87320300 -0.70838100
C -2.11071800 -1.90287400 -1.69399000
C -3.11012800 -0.94068800 -1.87462400
C -4.26263000 -0.94294200 -1.09755500
C -4.43833800 -1.91045100 -0.11053800
C -3.45705000 -2.87820600 0.07570200
H -1.55171300 -3.63644200 -0.54031900
H -2.96796400 -0.17275500 -2.62990400
H -5.02091000 -0.18215800 -1.25241100
H -5.33228800 -1.91024900 0.50312300
H -3.58507400 -3.64268200 0.83459800
C -0.88012600 -1.84507700 -2.57647000
H -0.65425600 -0.78821100 -2.73906100
H -1.12242700 -2.29341300 -3.54539500
C 0.37509900 -2.51925000 -2.01936200
H 1.16760900 -2.46167800 -2.76960700
H 0.20479600 -3.57850600 -1.80703100
C 0.84008000 -1.80946800 -0.76681100
H 1.05244400 -0.75742100 -0.97446900
H 0.09127000 -1.86915300 0.02562000
O 2.04008700 -2.49246800 -0.29933000
S 2.73392500 -1.89653600 1.00997200
O 3.78968700 -2.82959200 1.32555400
O 1.70310200 -1.61017400 1.99118000
C 3.43355400 -0.36902700 0.45827300
C 4.65368200 -0.39800900 -0.21369600
C 2.74696100 0.81855600 0.67230200
C 5.19339100 0.79512700 -0.66395700
H 5.17245500 -1.33687900 -0.36712400
C 3.30722900 2.00500700 0.21294800
H 1.79885100 0.81974400 1.19622300
C 4.52989000 2.01081100 -0.45716600
H 6.14506400 0.78843500 -1.18464000
H 2.78045600 2.93832200 0.37432900
C 5.13653300 3.29289700 -0.95513600
H 5.27514200 3.25662900 -2.03859500
H 4.50369800 4.14832400 -0.71772100
H 6.11960300 3.45425200 -0.50537400

(b)

C 3.23477400 1.66719400 -1.62556200
C 2.69906000 2.76285200 -1.02511400
C 1.03672100 1.39640000 -1.53584500
N 2.17849000 0.83543000 -1.93594500
H 4.24867000 1.32681100 -1.82189200
H 3.16260000 3.64687800 -0.62042900
H 0.01161900 1.01729000 -1.59314400
N 1.33402700 2.57139800 -0.98104300
C 2.31767300 -0.42435100 -2.66727700
H 1.93393500 -0.29953700 -3.67958600
H 3.38304900 -0.65328400 -2.69769200
H 1.75532000 -1.19922200 -2.15018800
C 0.33991400 3.52797800 -0.47317000
H -0.61919700 3.00893600 -0.53807700
H 0.57604300 3.72839700 0.57336800
C 0.35553500 4.81880700 -1.28964800
H -0.40871300 5.47631700 -0.86299500
H 1.31830700 5.32148100 -1.15383900
C 0.08217700 4.60668000 -2.77951400
H 0.19196900 5.56813400 -3.28932100
H 0.85187200 3.94940400 -3.20001400
C -1.30448900 4.02926200 -3.05936600
H -1.46687500 3.91449200 -4.13387200
H -1.44638400 3.05321200 -2.58586100
H -2.08075400 4.69919400 -2.67574000
F -1.72677300 1.32994400 -1.18331000
C -4.03671600 1.90404400 1.05970100
C -3.48872600 1.95632900 2.34613200
C -4.34875300 2.02091900 3.44351000
C -5.72978300 2.03534500 3.26621200
C -6.26774400 1.98371100 1.98387300
C -5.41715900 1.91603300 0.88286900
H -3.36970100 1.83168400 0.20096600
H -3.93214100 2.06483100 4.44554800
H -6.38460900 2.09014700 4.12874700
H -7.34256400 1.99675900 1.84319400
H -5.82958600 1.87351200 -0.11924700
C -1.99106400 1.90183800 2.54005000
H -1.71716000 2.45588700 3.44111100
H -1.50413500 2.38740000 1.68962100
C -1.44922200 0.46763200 2.65958300
H -1.85540600 -0.01858200 3.55161600
H -0.36087800 0.50327600 2.75911100
C -1.79935000 -0.33477500 1.42580500
H -2.86789900 -0.55325900 1.37656300
H -1.50037400 0.19177700 0.51248800

O -1.10846900 -1.62008800 1.51425000
S -0.77636700 -2.34999600 0.13376200
O -0.24396700 -3.63954000 0.52529900
O 0.05312900 -1.47042900 -0.67145500
C -2.34459400 -2.55436700 -0.65605200
C -3.00401100 -3.77058900 -0.52478200
C -2.89126200 -1.48225500 -1.36159500
C -4.24641800 -3.92064200 -1.12842300
H -2.54988800 -4.58455100 0.02702300
C -4.13773400 -1.65884400 -1.94492200
H -2.37557300 -0.52178600 -1.43050100
C -4.82884600 -2.87128500 -1.84140400
H -4.76978000 -4.86637900 -1.04334000
H -4.58545700 -0.83612200 -2.49290600
C -6.17687100 -3.02697400 -2.48888600
H -6.88622900 -2.30766000 -2.07161400
H -6.11162700 -2.83530800 -3.56275100
H -6.57533600 -4.03075100 -2.33988200
C 2.46625900 -2.15291500 1.51060100
C 2.96006000 -1.01520700 2.06512300
C 3.90650900 -1.27199900 0.08219900
N 3.07126100 -2.29175000 0.27957400
H 1.74090500 -2.86475500 1.86735800
H 2.75645700 -0.54282000 3.01155400
H 4.54513400 -1.03389000 -0.79822500
N 3.84822200 -0.48367400 1.15544100
C 2.84060000 -3.37857300 -0.66759400
H 2.99013300 -4.33095800 -0.16121800
H 1.82289300 -3.31888700 -1.04784100
H 3.55542400 -3.27697800 -1.48185800
C 4.70332500 0.69419700 1.34882400
H 5.14202700 0.90103700 0.37152300
H 4.06208600 1.52878700 1.63496800
C 5.77372700 0.42877200 2.40390800
H 6.35429000 1.35039900 2.51215200
H 5.28762000 0.24135200 3.36651500
C 6.70618000 -0.73120200 2.05082000
H 7.39606800 -0.88038100 2.88616700
H 6.12385500 -1.65615900 1.96906500
C 7.49929900 -0.49935200 0.76476100
H 8.19810400 -1.32111100 0.59048700
H 6.85527500 -0.41687400 -0.11664600
H 8.08515100 0.42245800 0.83942100
F 5.41892300 -0.14416800 -1.84223700
